# Rigorous Approach for Design of Differential Coupled-Line Directional Couplers Applicable in Integrated Circuits and Substrate-Embedded Networks

**DOI:** 10.1038/srep25071

**Published:** 2016-04-26

**Authors:** Kamil Staszek, Krzysztof Wincza, Slawomir Gruszczynski

**Affiliations:** 1 AGH University of Science and Technology, Department of Electronics, Krakow, 30-059, Poland

## Abstract

We report rigorous approach for the design of differential coupled-line directional couplers in multilayer dielectric structures. In the proposed procedure numerically calculated per-unit-length parameters of coupled transmission lines are utilized for derivation of differential couplers’ properties. The known description technique with multimode scattering parameters has been extended to the eight-ports considered in the paper and the properties resulting from symmetry of the considered networks have been shown. Exemplary 3-dB and 8-dB coupled-line directional couplers have been designed and experimentally evaluated. Nodal to mixed-mode conversion of scattering parameters has been applied to allow for measurements of the physically realized models. Results of measurements are shown to confirm the presented theoretical considerations.

Directional couplers have been for years utilized as basic components in high-frequency electronics in different technologies including monolithic or dielectric-substrate-embedded^1^,^2^,^3^,^4^,^5^,^6^,^7^,^8^,^9^. Depending on application, different types of couplers have been developed and realized in various techniques. Among known directional couplers, coupled-line couplers constitute an important group offering wide operational bandwidth with relatively small overall dimensions. The coupled-line directional couplers can cover a wide range of coupling values from very week couplings for microwave power monitoring applications to equal-split directional couplers for signal distribution in antenna networks, balanced circuits, mixer applications, etc. The design of coupled-line directional couplers having strong coupling requires, however, a multilayer technology to allow for sufficient mutual coupling of the coupler’s conductors. The realization of such couplers has been widely described in a number of papers, where different dielectric structures as well as different topologies have been considered^3^,^4^.

The majority of the reported work focuses on the development of directional couplers operating with nodal excitations of their coupled conductors^2^,^8^. Nowadays it is common, however, to design analog electronic circuits having differential inputs and outputs due to their superior interference rejection. Many examples of differential amplifiers, mixers, filters can be found in literature^10^,^11^,^12^,^13^,^14^. Following the development of differential circuits, methods for their description have been proposed, in particular mixed-mode scattering parameters have been introduced, where differential, common and mixed S parameters have been defined^15^,^16^,^17^,^18^. Following the recent trend of differential circuits’ utilization, also directional couplers operating in differential mode have been recently described^19^. In such directional couplers the proper characteristics are obtained for differential mode only.

In this paper a rigorous method for the design of differential mode directional couplers has been proposed. Such couplers can find applications in interconnecting surface-mount integrated circuits. Therefore, the most interesting are the directional couplers having coupled-lines embedded in the dielectric (applicable in multilayer monolithic, PCB or LTCC technology), which are discussed in this paper. In the proposed method of directional couplers’ design an approach based on calculation of capacitive matrix of a coupled-conductor system and its appropriate reduction has been used, from which the final derivation of coupler’s impedance and coupling coefficient can be made. The numerically found coupled-line geometry can be further utilized in electromagnetic simulations where additional effects as losses and parasitics associated with signal line connections can be taken into account. The presented in this paper approach has not been reported so far with respect to the differential couplers’ design, in^19^ only a simplified design approach has been proposed, where the existence of a ground-plane has been neglected. Such a simplification was justified by the geometry of the considered coupled lines where the distance between coupled strips and a ground plane was relatively large. In contrary, the couple-line geometry considered in this paper does not allow one to make similar simplifications, therefore, a strict approach has been presented. Further, design of an exemplary equal-split directional coupler has been shown following the proposed procedure. Moreover, a recently introduced mixed-mode scattering matrix, developed for the description of four-port networks has been extended on the considered in this paper eight-ports, allowing for calculation of mixed-mode scattering parameters from nodal simulation and measurement results. To validate the proposed design technique of differential-mode directional couplers, 3-dB and 8-dB directional couplers have been manufactured. The nodal measurements of the developed couplers have been made and the obtained results have been converted with the described procedure into differential and common mode S parameters.

## Methods

### Theoretical Analysis

**Figure 1:**
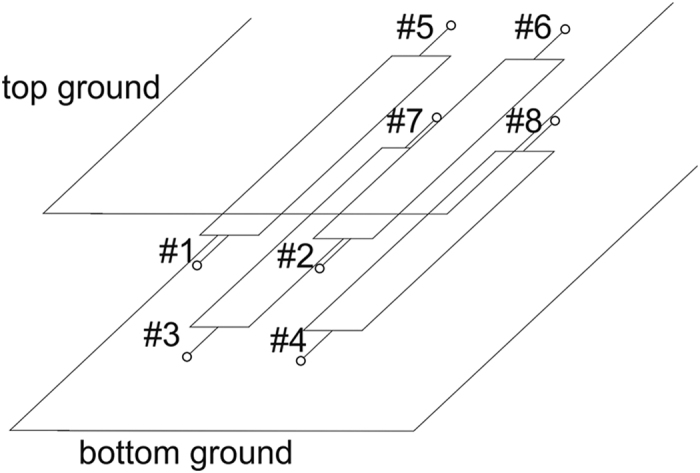
A generic view of a four-conductor coupled-line system placed in between two ground planes with the proposed terminal labeling.

**Figure 2:**
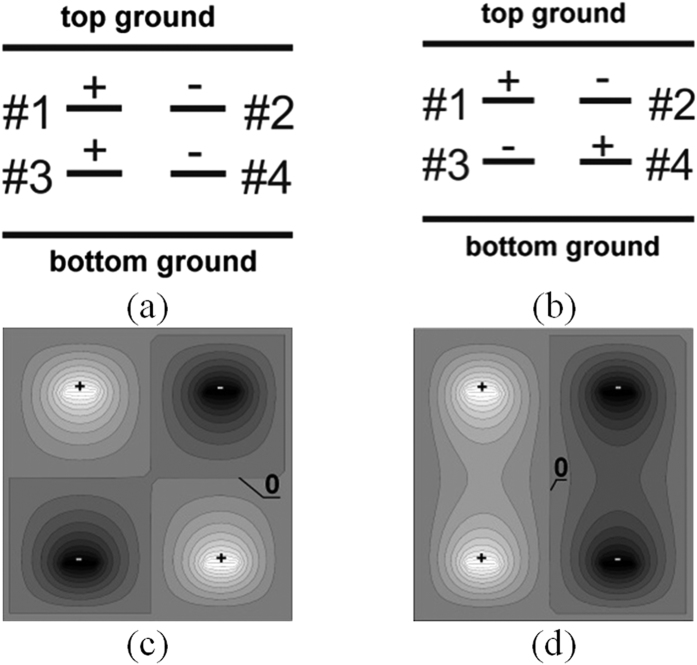
Cross-sectional view of a four-strip symmetric-coupled-line geometry with excitations of even-differential (**a**) and odd-differential (**b**) modes and corresponding respective potential distributions (**c**,**d**).

A generic view of a multilayer stripline couple-line system considered in the paper is shown in Fig. 1, and consists of four equal-width coupled conductors. The conductors are placed in homogeneous dielectric medium between two ground planes, upper and lower, what ensures full symmetry of the system and allows for achieving good isolation and directivity of the directional coupler. In Fig. 1 also port numbers are assigned consistent throughout the paper. In the design of balanced directional couplers there is more than one possibility of choosing pairs of conductors constituting balanced lines. For the analysis it is assumed that one of the balanced lines has terminals #1,#2, #5 and #6, whereas the second line has the remaining terminals #3, #4, #7 and #8. Since the considered structure is a symmetrical one, a well-known modal approach can be applied in the proposed coupler, however, one can distinguish four modes instead of two, i.e. even-differential and odd-differential modes as well as even-common and odd-common modes. Figure 2 shows the appropriate excitations of coupled conductors for both differential modes existing in a symmetric coupled-line system, also corresponding potential distributions for both excitations are presented, obtained by numerical solving of Laplace equation using the finite difference method for the given boundary conditions.

**Figure 3:**
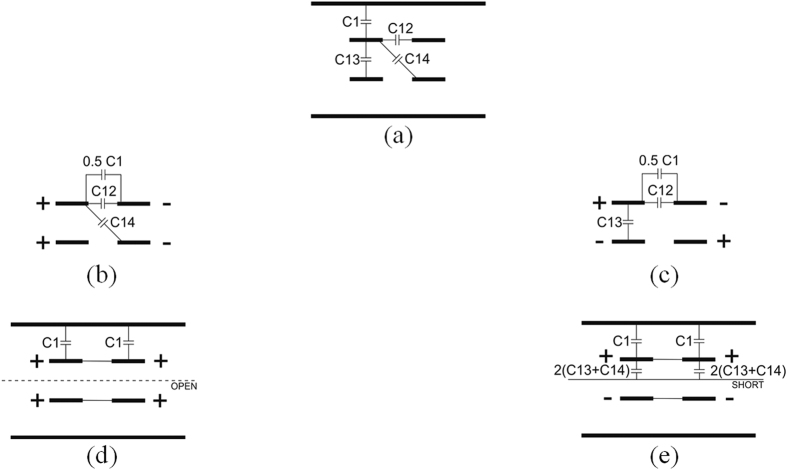
Capacitive elements related to a particular conductor of the considered four-strip symmetric-coupled-line geometry (**a**) and the equivalent capacitive elements related to even-differential (**b**), odd-differential (**c**), even-common (**d**) and odd-common (**e**) modes.

Having defined the geometry of coupled lines one can calculate its per-unit-length parameters, i.e. capacitance and inductance matrices. The resulting parameters of the directional coupler for differential (and also common) mode can be calculated after appropriate reduction of the capacitance matrix. In Fig. 3 the capacitive elements associated with one of the conductors are presented. Assuming the symmetry of the structure identical four capacitive elements are associated with each of the remaining three conductors. Since the differential excitation is of the primary interest, the reduced per-unit-length capacitances for that mode are shown in Fig. 3b,c, and it is seen that for even-differential mode the capacitance is equal to:


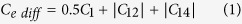


And for the odd-differential mode the capacitance is equal to:


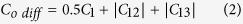


where *C*_1_ = *C*_11_–|*C*_12_|–|*C*_13_|–|*C*_14_|.

For completeness the even- and odd-common mode capacitances are expressed respectively as (Fig. 3d,e):


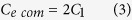



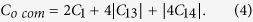


Assuming homogeneous dielectric filling of the coupled-line geometry the modal impedances can be expressed as:


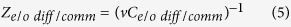


where *v* is a phase velocity calculated as 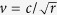 (*c*–the free space speed of light, *ε*_*r*_ − electric permittivity of the dielectric substrate).

### Directional Coupler Design

**Figure 4:**
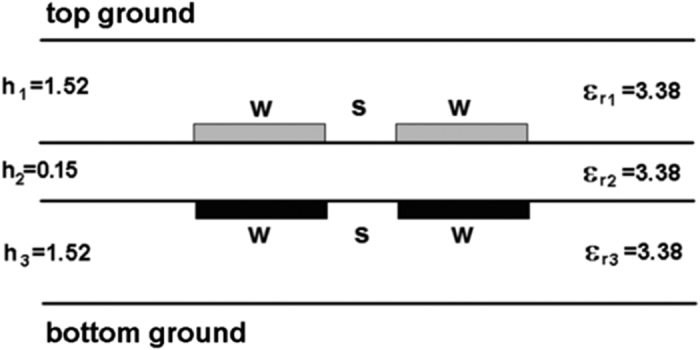
Cross-sectional view of the dielectric structure used for the design of the proposed differential directional couplers.

The presented in Section II procedure has been utilized for the design of a coupled-line differential directional coupler in a dielectric structure shown in Fig. 4 using Arlon 25N laminates. The capacitive matrix of the coupled conductors has been calculated numerically with a *Linpar* software^20^ and the results have been used to calculate differential coupler’s properties. The final dimensions have been found in an iterative process in which for each iteration the dimensions were modified manually and the parameters of the coupler were found. In order to automate the calculation process a dedicated calculator has been created which reads the *Linpar* results and recalculates the parameters with the use of the equations (1. Assuming the terminating impedance of the coupled lines for differential modes *Z*_0_ = 50 Ω, the final widths of the strips have been found to be *w* = 1.21 mm and the spacing between conductors equals *s* = 0.2 mm. The calculated capacitance matrix equals:


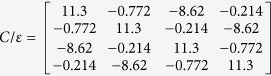


The equivalent capacitance matrix for a directional coupler operating in the differential mode, calculated taking into account eqs (1 and 2) equals:


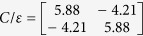


From which one can easily find the terminating impedance of the coupled lines *Z*_0_ = 49.92 Ω, the modal impedances *Z*_0*e diff*_ = 122.78 Ω, *Z*_0*o diff*_ = 20.3 Ω and the coupling coefficient of the coupled lines equals *k* = 0.7162 (*C* = −2.9 dB). For the common mode the following values have been found based on (3) and (4):


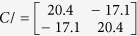


*Z*_0_ = 20.09 Ω, the modal impedances *Z*_0*e com*_ = 75.15 Ω, *Z*_0*o com*_ = 5.37 Ω and the coupling coefficient of the coupled lines equals *k* = 0.866 (*C* = −1.24 dB).

As it is seen from the obtained values, the designed coupled-line geometry constitutes an equal-split coupled-line directional coupler for the differential mode, and its impedance match equals *Z*_0_ ≅ 50 Ω.

**Figure 5:**
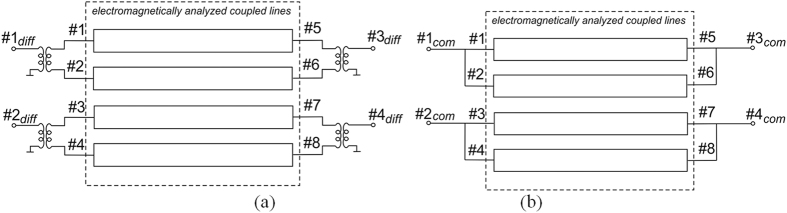
Schematic diagram of the analyzed four-strip coupled-line directional coupler for differential (**a**) and common (**b**) excitations.

**Figure 6:**
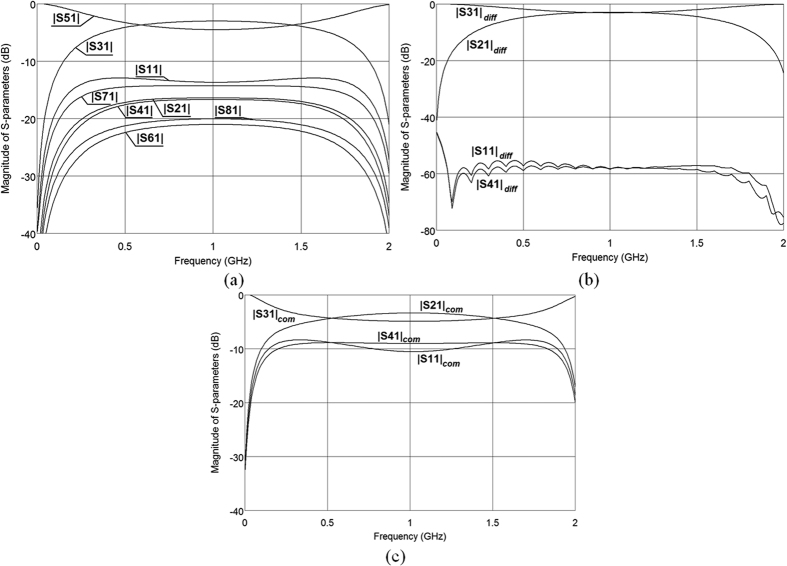
Electromagnetically calculated S-parameters of a four-coupled-strip system for excitations: nodal (**a**), differential (**b**) and common (**c**). The normalizing impedance for all three sets of S-parameters equals *Z*_0_ = 50 Ω.

Initial verification of the coupled line geometry has been made by means of electromagnetic calculations of the designed structure in *AWR Microwave Office* software. The schematic diagrams of the considered circuits are presented in Fig. 5, where a box consisting of electromagnetically calculated S-parameters of four-coupled-strip geometry has been excited by means of ideal transformers for the differential mode and by direct connection of strips for the common mode. The calculated S-parameters are presented in Fig. 6 (assuming normalizing impedance *Z*_0_ = 50 Ω for all three cases), where general S-parameters for nodal excitation as well as S-parameters for differential and common modes are shown. As it is seen the designed coupled-line geometry can be successfully used as a directional coupler for the case of differential excitation and the terminating impedance equals *Z*_0_ = 50 Ω for which the return losses and isolation are ideal. The obtained coupling equals C = −2.93 dB and is in full agreement with the theoretical predictions shown in Section II. Additionally, the coupling characteristics of the differential coupled-line directional coupler shown in Fig. 6c have been compared with the theoretical ones given in^1^, and an excellent agreement has been obtained confirming that the proposed network features the properties of an ideal coupled-line directional coupler.

**Figure 7:**
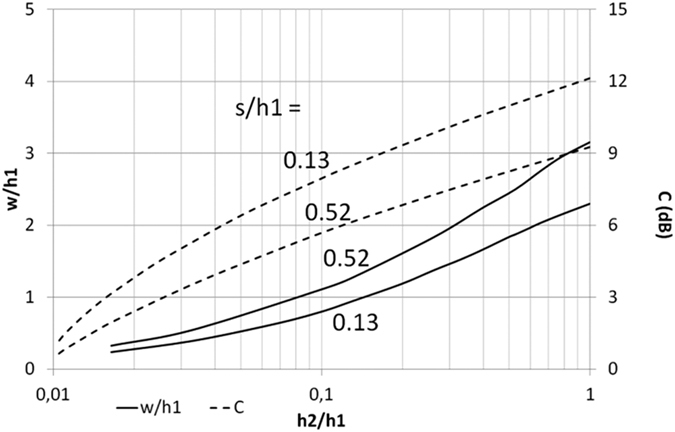
Calculated conductor width *w*/*h*_1_ and coupling *C* for the coupled-line geometry shown in Fig. 4 for the assumed differential terminating impedance *Z*_0_ = 50 Ω and two slot dimensions.

Following the presented design procedure different single-section directional couplers in the proposed coupled-line geometry can be designed. In particular different coupling coefficients between differentially fed coupled lines can be realized by adjusting the ratio of inner to outer layer thicknesses *h*_2_/*h*_1,3_ (*h*_1_ = *h*_3_). The results of calculations of normalized width *w*/*h*_1_ and coupling vs. the layers’ thicknesses ratio *h*_2_/*h*_1_ have been shown in Fig. 7. As it is seen the coupling of the directional coupler can be changed in a wide range from 1 dB to 12.1 dB for a range of thicknesses ratio *h*_2_/*h*_1,3_ from 0.016 to 1.

### Mixed-mode Scattering Matrix

Since the differential-mode circuits became practically used, their description in terms of scattering parameters appeared in a number of papers, and as a result mixed-mode S-parameters have been introduced^15^,^16^,^17^,^18^. Since in all the reported cases the considerations were limited to four-port nodal networks reducing to two-port differential networks, it is therefore justified for completeness to briefly extend the mixed-mode S-parameters’ formulation to the considered here eight-ports, which reduce in turn to four-port differential networks. This is in particular important for experimental investigation, since only nodal parameters can be measured with standard measurement techniques, and the mixed-mode S-parameters need to be extracted from the measurements. Following the derivation shown in^15^, the mixed mode scattering matrix can be expressed as:


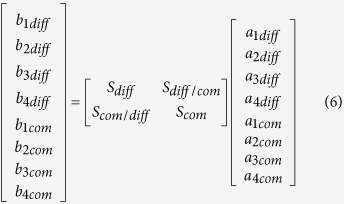


The normalized power waves, for the chosen port indexing and the choice of differential and common excitations, are defined as follows:


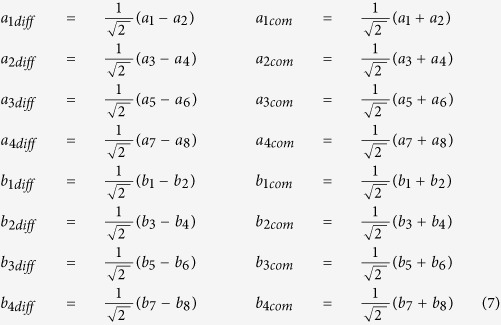


The mixed-mode sub-matrices (6) can be expressed as:


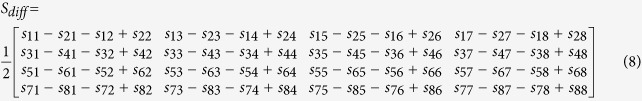



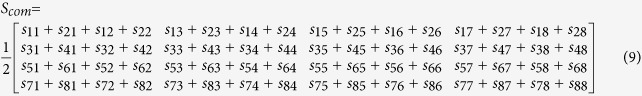



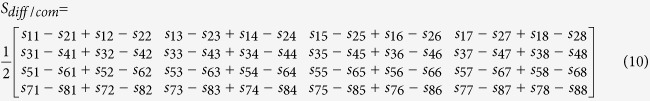



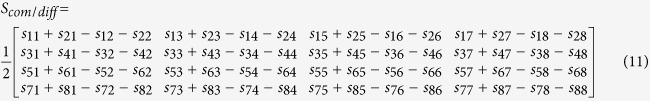


and can be calculated as^16^:


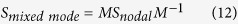


where M equals:


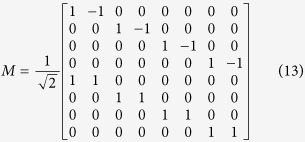


and *S*_*nodal*_ is a classic scattering matrix with signals at all ports referenced to the common potential.

Having the mixed-mode scattering matrix explicitly given, it can be shown that for a double symmetrical passive network all the elements of sub-matrices (12) and (13) equal zero. From the symmetry of the network one can write:


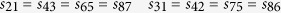



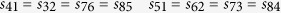



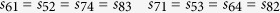



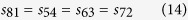



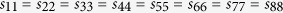


Additionally from the reciprocity we have:


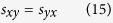


By applying (14) and (15) to (12) and (13) one can easily prove that:


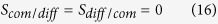


It is important to underline that (16) confirms no conversion between common and differential modes in a double symmetrical eight-port having four differential ports. Hence, the Common-Mode Rejection Ratio (CMRR_D/CtoD_), defined as differential-mode gain to the conversion gain from common to differential modes after^21^, is theoretically infinite since the second term equals 0. It can be said, therefore, that such circuits do not contribute to the propagation of interferences, which are induced in all lines with equal amplitudes and phases (common-mode signals) and no conversion of such signals to the utilized differential modes occurs.

## Results

**Figure 8:**
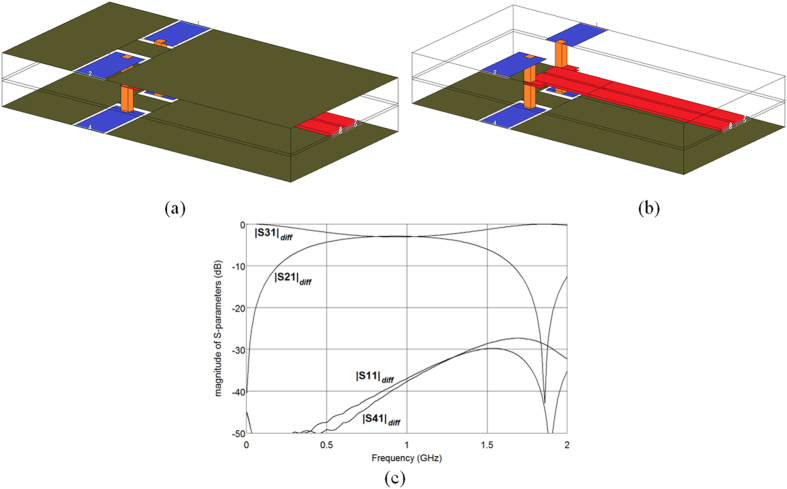
3D view of the modeled directional coupler using AWR Microwave Office simulator showing the connections between the coupled lines and the sections of coplanar waveguides placed at the top and bottom ground planes (**a**), the same view with the top ground plane removed for detailed visibility of the structures (**b**) and electromagnetically calculated S-parameters of the designed 3-dB differential directional coupler for differential excitation (**c**).

**Figure 9:**
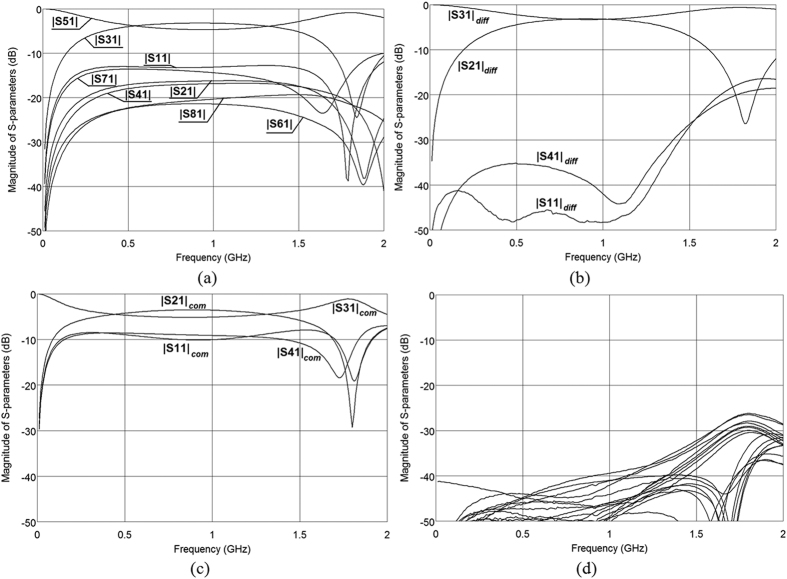
Measured S-parameters of the designed four-coupled-strip geometry of a 3-dB differential directional coupler for nodal (**a**), differential (**b**) common (**c**) and common/differential (**d**) excitations. The normalizing impedance equals *Z*_0_ = 50 Ω in all cases.

**Figure 10:**
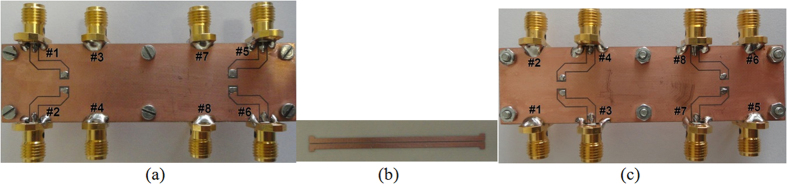
Photograph of the developed model of the 3-dB differential coupled-line directional coupler. Top ground with sections of coplanar waveguides and SMA connectors (**a**), inner laminate layer with coupled conductors (**b**) and bottom ground with sections of coplanar waveguides and SMA connectors (**c**).

To verify experimentally the proposed design method, a 3-dB differential coupler described in Section III has been manufactured and measured. Prior to the manufacturing the problem of connections between the coupled strips and the coaxial SMA connectors has been resolved. In order to apply coaxial connectors, coupled strips have been connected to short sections of coplanar waveguides placed at the top and bottom ground planes by means of via connections. The dimensions of the applied coplanar waveguide sections are as follows: strip widths *w*_*t*_ = 2 mm, slot widths between strip and ground plane *s*_*t*_ = 0.2 mm, via diameters *d*_*t*_ = 0.5 mm. Figure 8a shows 3D view of the modeled directional coupler using AWR Microwave Office simulator showing the connections between the coupled lines and the sections of coplanar waveguides (for better view in Fig. 8b the same structure has been shown with the top ground plane removed). The entire differential directional coupler has been analyzed electromagnetically to confirm the influence of the transitions between coupled lines and coplanar waveguides. The results of calculations for the differential excitation are shown in Fig. 8c and the obtained results are in agreement with the electromagnetic calculations of the coupled-lines only. In this case the applied transitions degrade the return losses and isolations of the directional coupler but still both characteristics are better than 30 dB. In this case the center frequency of the directional coupler has shifted down due to the fact that the added transitions increased the lengths of the coupled lines. For final verification the designed coupler has been fabricated, the laminate layers have been assembled mechanically together to provide a dielectrically homogeneous structure. The measurements have been made with a vector network analyzer calibrated at its ports (SMA standard). Subsequently, the short sections of coplanar waveguides and SMA connectors have been de-embedded to retrieve the parameters of the coupled-lines. The measurement results have been shown in Fig. 9. As it is seen a very good agreement has been obtained between measurements and theoretical calculations, the measured responses are shifted in frequency by about 5% with respect to the characteristics from Fig. 6 which is caused by the application of transitions between the coupled lines and coplanar waveguides and the frequency shift is small with respect to the coupler’s bandwidth which equals ∼65%. In particular, the designed four-strip coupled-line geometry constitutes a high-performance coupled-line 3-dB differential directional coupler having impedance match and isolation better than 30 dB. Moreover, it has been verified that for the symmetric structures no conversion between common and differential mode exists. In the measured case the conversion losses from common to differential modes are on the level of 50 dB, hence, the CMRR_D/CtoD_ ≈ 45 dB. Figure 10 presents the photograph of the manufactured coupler where the short sections of coplanar waveguides are visible as well as the coupled strips of symmetric coupled-line geometry. It has to be commented that in future applications the via connections can lead directly to the top layer and connect the strips to the pads, where integrated circuits with symmetric input/output ports are applied.

**Figure 11:**
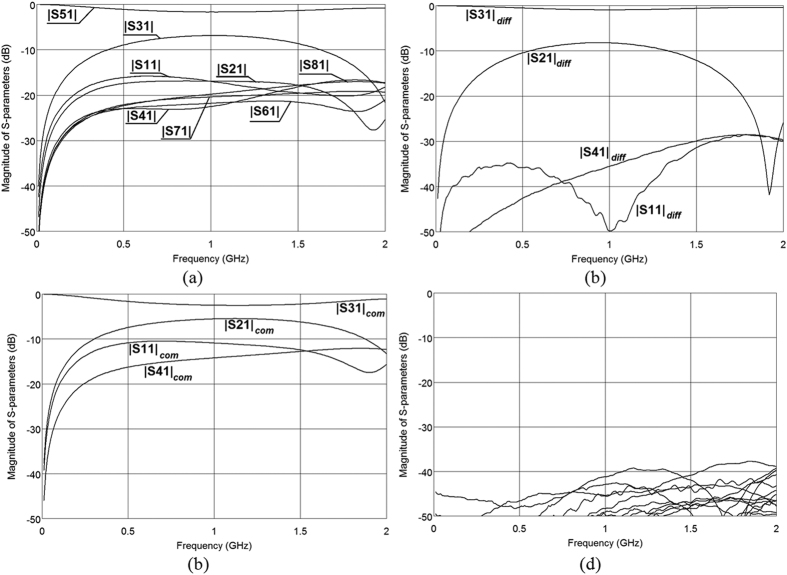
Measured S-parameters of the designed four-coupled-strip geometry of an 8-dB differential directional coupler for nodal (**a**), differential (**b**) common (**c**) and common/differential (**d**) excitations. The normalizing impedance equals *Z*_0_ = 50 Ω in all cases.

To show the possibility of designing directional couplers having weaker coupling an 8-dB directional coupler has been designed following the procedure described for the presented 3-dB differential directional coupler. The dimensions of the coupler have been taken from Fig. 7 and assuming the same thicknesses of the top and bottom layers h_1,3_ = 1.52 mm, the thickness of the middle layer equals h_2_ = 0.76 mm, and the width of the coupled lines equals w = 2.8 mm. The measurement results of the designed 8-dB differential coupled-line directional coupler are shown in Fig. 11. As it is seen the designed differential coupler features return losses and isolation better than 30 dB in a broad frequency range. The worst case conversion between differential and common modes is as good as 40 dB (*S*_11*diff/com*_) and the resulting CMRR_D/CtoD_ ≈ 55 dB for transmission (*S*_13*diff*_/*S*_13*diff/com*_) and CMRR_D/CtoD_ ≈ 42 dB for coupling (*S*_12*diff*_/*S*_12*diff/com*_).

## Discussion

The obtained results in case of differential coupled-line directional couplers are comparable to the results of otherwise reported single-ended directional couplers designed in stripline technologies^2^,^3^,^7^. The measured return losses are as good as 30 dB are typically considered as a top quality. The presented coupler can be directly compared with the coupler reported in^19^, since both couplers operate in differential modes. The coupler from^19^ features poorer return losses of about 15 dB and isolation on the level of 22 dB, however, it operates at higher frequency *f*_0_ = 30 GHz, moreover, it is realized in monolithic technology and in inhomogeneous dielectric structure. All the facts can degrade the performance of the coupler presented in^19^. Nevertheless, the experimental results obtained in our investigation prove the usefulness of the proposed rigorous design procedure of substrate-embedded differential directional couplers and can be applied in the development of modern integrated circuits where many differentially driven building blocks may be interconnected with the proposed couplers.

Finally it is worth to comment on the robustness of the couplers performance against the manufacturing errors and material uncertainties. The most significant errors in manufacturing are related to the etching accuracy of the photolithographic processes which are on the level of ± 10 μm. Significant errors related to material parameters uncertainties are associated with the dielectric constant characterization, and typically such uncertainties are below 5%. To investigate the influence of both sources of errors on the proposed 3-dB directional coupler’s properties, electromagnetic simulations have been performed in which the widths of coupled lines have been increased by 20 μm, and the dielectric constant of the material has been decreased by 5%. The obtained results have shown that the manufacturing accuracy has negligible influence on the coupler’s parameters since the dimensions of the proposed structure are by far greater than the etching accuracy. The influence of the dielectric constant changes have more distinct influence on the directional coupler’s return losses and isolations, and the change of dielectric constant by 5% can degrade both by about 4 dB.

## Conclusions

A method for the design of differential coupled-line directional couplers has been proposed. In the design a numerical calculation of per-unit-length parameters has been used, from which the properties of differential couplers have been calculated. The designed coupled-line geometry has been verified by means of electromagnetic simulations. As it has been shown such a coupled-line system constitutes a 3-dB differential coupled-line directional coupler. Moreover, its properties for the common mode have been shown and are in agreement with the theoretical predictions. Further, the known mixed-mode S-parameters utilized for the description of a four-port network have been extended on the considered eight-ports, which has allowed to evaluate the measurement data. Also the property of mode conversion has been commented confirming that in case of symmetrical eight-ports no conversion between differential and common modes occurs. Finally, the designed 3-dB coupled-line differential directional coupler has been manufactured and measured. The measurement results have fully proven the correctness of the design method. The measured differential directional coupler features equal power split between direct and coupled ports, the isolation better than 30 dB and return losses better than 40 dB. Moreover, the measured conversion losses between differential and common modes are as high as 40 dB. Additionally, the designed method of the differential coupled-line directional couplers has been verified by the design of an 8-dB coupler. Also in this case the agreement between theoretical and measured characteristics is very good and the coupler features high conversion losses between differential and common modes.

## Additional Information

**How to cite this article**: Staszek, K. *et al.* Rigorous Approach for Design of Differential Coupled-Line Directional Couplers Applicable in Integrated Circuits and Substrate-Embedded Networks. *Sci. Rep.* **6**, 25071; doi: 10.1038/srep25071 (2016).
